# Autism Spectrum Disorder Traits Predict Interoceptive Deficits and Eating Disorder Symptomatology in Children and Adolescents with Anorexia Nervosa—A Cross-Sectional Analysis: Italian Preliminary Data

**DOI:** 10.3390/pediatric16040092

**Published:** 2024-12-05

**Authors:** Maria Califano, Jacopo Pruccoli, Melania Martucci, Caterina Visconti, Emma Barasciutti, Carla Sogos, Antonia Parmeggiani

**Affiliations:** 1 IRCCS Istituto delle Scienze Neurologiche di Bologna, UOC Neuropsichiatria dell’Età Pediatrica, Centro Regionale per i Disturbi della Nutrizione e dell’Alimentazione in età evolutiva, 40139 Bologna, Italy; maria.califano3@studio.unibo.it (M.C.); jacopo.pruccoli@gmail.com (J.P.); caterina.visconti@studio.unibo.it (C.V.); 2 Dipartimento di Scienze Mediche e Chirurgiche (DIMEC), Università di Bologna, 40126 Bologna, Italy; emma.barasciutti@studio.unibo.it; 3 Child Neuropsychiatry Unit, Department of Human Neurosciences, Sapienza University of Rome, 00185 Rome, Italy; melania.martucci@uniroma1.it (M.M.); carla.sogos@uniroma1.it (C.S.)

**Keywords:** feeding and eating disorders, anorexia nervosa, autism spectrum disorder, interoceptive deficits, alexithymia

## Abstract

Background: Anorexia Nervosa (AN) is a severe Feeding and Eating Disorder (FED) that is more prevalent in females, often manifesting during adolescence. Recent research highlights an elevated presence of comorbid Autism Spectrum Disorder (ASD) traits among individuals with AN, with specific expressions in females accounting for sensorial and interoceptive experiences. This study retrospectively explores the association between ASD traits, eating symptomatology, and interoceptive deficits in Italian female adolescents with AN. Methods: A retrospective evaluation of female AN/Atypical AN patients (n = 52) aged 13–17 years was conducted at two university pediatric hospitals in Italy. The participants underwent neuropsychiatric assessments, including the Autism Diagnostic Observation Schedule-Second Edition (ADOS-2), and measurement of ASD traits with the Autism-spectrum quotient (AQ), camouflaging ASD traits Questionnaire (CAT-Q), Toronto Alexithymia Scale (TAS-20), and FED-symptomatology-related measures. Results: Overall, 9.6% of the participants exhibited an ADOS-2 clinical impression consistent with ASD. Higher scores in AQ and CAT-Q revealed ASD traits and camouflaging strategies. The interoceptive deficits positively correlated with the ASD traits, alexithymia, and camouflage, and TAS—Difficulty Identifying Feelings emerged as the sole predictor for interoceptive deficits. Discussion: This Italian study preliminarily underscores the importance of recognizing ASD traits in the AN population, emphasizing early intervention strategies. The intersection of alexithymia and interoceptive deficits emerges as a crucial nexus between ASD and AN, with potential therapeutic implications.

## 1. Introduction

Anorexia Nervosa (AN), a severe Feeding and Eating Disorder (FED) characterized by low body weight, restricted caloric intake, obsessive fear of gaining weight, and disturbance in the experience of the body shape, is far more common in females than in males and typically manifests during adolescence [1]. In worldwide studies, AN has a lifetime prevalence of 0.8–6.3% among women and 0.1–0.3% among men [2]. Autism Spectrum Disorder (ASD), on the other hand, is a Neurodevelopmental Disorder with an estimated prevalence of 100 out of 10,000 children in the population [3], with a male-to-female ratio of 4:1 [4]. In the last decade, research has documented an elevated presence of comorbid autistic symptoms among people with AN [5,6] and specific similarities both in terms of neuropsychological profiles and socioemotional difficulties [5] Based on the latest review on the topic by Westwood and Tchanturia [5], studies report an over-representation of ASD symptoms in AN patients, with estimates of 20% to 30% of women in treatment for AN meeting the clinical cut-off for ASD. It is important to consider that, in the last two decades, with the broader interest in a dimensional approach to psychopathology, the literature has focused on investigating the presence not only of ASD but also of subthreshold ASD traits among the clinical and general populations [7,8]. The term “ASD traits” identifies a set of characteristics that can be framed within the Broader Autism Phenotype (BAP), i.e., the set of symptoms similar to those typical of ASD, such as emotional detachment, difficulties in social relationships, expression of emotions, restricted or atypical interests, and presence of language and communication deficits. Such characteristics, usually found in relatives of people with ASD, are more subtle in expression and of subclinical interest and are not usually associated with relevant functional impairment [9,10,11,12,13,14,15]. In recent studies, subthreshold ASD traits have gained relevance since they seem to be associated with an increase in vulnerability towards the development of other psychiatric disorders such as AN [16,17]. The overlap of ASD traits and AN leads to more serious psychopathological impairment characterized by inflexibility, ritualistic behaviors, mood disorders, phobias, and social impairment [18]. In this regard, a relevant issue is the conceptualization of AN as a gender-specific presentation of ASD, which may explain the opposite gender differences in ASD and AN prevalence. It seems that female patients could recognize their social difficulties and mask them, often by assimilation of others’ behaviors and habits. Moreover, the pattern of narrow interests and repetitive behaviors amongst females with ASD consists of more socially accepted topics, such as spending time with animals, reading fiction, or dietary habits.

Hence, this specific female presentation of ASD could result in missed early diagnosis, which may be recognized in adolescents affected by AN.

Several studies underline how closely interconnected ASD and eating symptoms are, even in the experience of patients. In particular, Kinnaird et al. interviewed nine women diagnosed with ASD and four women with high ASD traits about their experiences regarding AN and the treatment they received [19,20]. The participants reported experiencing how ASD traits motivate different eating difficulties in manners that are not considered in the traditional models of AN. The rigidity and inflexibility associated with ASD symptoms seem to contribute significantly to the development of fixed routines and rituals concerning food. It is relevant that issues such as the desire to lose weight, low self-esteem, and difficulties related to one’s body image are less relevant in the development of an eating pathology compared to other less typical motivations, such as the need for control, sensory difficulties, organizational problems relating to cooking and purchasing food, physical exercise as a possibility of self-stimulation, and the polarization of thought regarding food issues, which acts as a narrow and absorbing interest [19,20]. A relevant topic in this field concerns an atypical sensorial profile and interoceptive deficits. Women with comorbid AN and ASD traits reported adverse sensory responses related to noise, touch, and some lights. Such sensory overload seems to lead to the choice of fasting as a way to alleviate the discomfort associated with the sensations described [20,21]. In this regard, the literature highlights how alexithymia with deficits in interoception could be an important connection point to explain the continuum between ASD and AN [21,22]. Interoception refers to the perception of a wide range of physical states in addition to emotions, including heart rate, respiratory effort, temperature, fatigue, hunger, thirst, satiety, muscle aches, pain, and itching [23]. Women affected by AN often report altered perceptions relating to the sense of hunger and satiety. The comorbidity with ASD traits seems to make internal sensations such as fullness after meals and the sensation of digesting food very annoying, with consequent food restriction [21,23]. Many other patients, however, have reported hyposensitivity to internal sensations. This entails difficulty in recognizing and decoding one’s emotional states, as well as in interpreting those sensations that are related to food, such as hunger and satiety. Some women reported consistently forgetting meals because they did not realize they were hungry. Others said they ate too much without realizing it and then consequently limited their food intake. Interoceptive difficulties seem to be connected to dysfunctional eating behaviors, such as limiting food intake for long periods or entering a cycle of binge eating and restriction [23]. Some women suggest that they had never been able to regulate their eating routine without relying on external signals, such as the time of day or the size of a plate, even before AN. Several health professionals have pointed out that these characteristics are connected to ASD or ASD traits. In fact, girls without ASD traits, at least in the first phase, feel hungry but actively contrast this sensation. Girls with ASD traits or a diagnosis of ASD, instead, seem to present a different sensory profile in this sense, even before the onset of the disorder [23]. Recent studies that propose a model of alexithymia, rather than being associated only with poor decoding of affective states, as a general failure of interoception mechanisms, could provide some explanations regarding the affinities between the two disorders [23]. A recent systematic review, in fact, suggests how identifying a common path between AN, ASD, and alexithymia with a dimensional approach can more specifically characterize some clinical phenotypes of AN [24]. Exploring this aspect could clarify whether the deficits in decoding one’s internal affective and non-affective states and the atypical sensory aspects, common to the two disorders, can explain the difficulties in managing internal bodily sensations and the perception of one’s own bodily boundaries, which are less certain regarding the typical symptoms of FED [25].

Hence, the aim of our study has been to investigate the presence of ASD traits or comorbidity with ASD in patients diagnosed with AN in the developmental age range in two Italian centers with competence regarding ASD and eating disorders and to investigate the possible associations between ASD traits and the variables involved in the evaluation of eating symptomatology and interoceptive deficits in a sample of patients with AN.

## 2. Materials and Methods

### 2.1. Design

This was a preliminary retrospective evaluation of patients affected by AN. The study expanded a previous study assessing correlations between ASD traits and FED symptoms in young patients with AN [26]. The study was conducted according to the ethical standards and principles regulating clinical investigations and consistent with the Helsinki Declaration of 1975, as revised in 2008.

### 2.2. Participants

Study participants presented the following inclusion criteria:

1. Female patients;
2. Age between 13 years, 0 months and 17 years, 11 months;
3. A diagnosis of AN (DSM-5) or Atypical Anorexia Nervosa (AAN) performed at the FED services of the centers involved in the study;
4. Elapsed time of at least one month since the last psychopharmacological therapy (antidepressants, mood stabilizers, antipsychotics, or anxiolytics) was introduced;
5. Body Mass Index (BMI) > 14.00 kg/m^2^;
6. Acquisition of informed consent.

Patients for whom it was not possible to provide sufficient clinical/therapeutic data were excluded.

### 2.3. Neuropsychiatric Assessment

Demographics and clinical data were collected, including age, family psychiatric history, pharmacological therapy, the presence of comorbidities, and BMI at the time of evaluation. The neuropsychiatric assessment included the following:

- Autism Diagnostic Observation Schedule-Second Edition [27]: The ADOS-2 represents the gold standard test for the diagnosis of ASD. Module 4 is used to evaluate adolescents and adults without language disorders. It consists of scores in the areas of communication, reciprocal social interaction, imagination and creativity, stereotyped behavior, and restricted interests. The scores in the areas of communication and reciprocal social interaction are then used to obtain a final score that can correspond to a diagnosis of “Autism”, “Autism Spectrum”, or “Non-Spectrum”.
- Autism-spectrum quotient (AQ) [28]: This allows us to evaluate the presence of ASD traits in adolescents and adults. It consists of five different areas: social skills, attention shifting, attention to detail, communication, and imagination. Two different versions of the AQ were used in this study: AQ 12–15 years, administered to parents, and AQ 16+ years, which is a self-administered test.
- Camouflaging Autistic Traits Questionnaire (CAT-Q) [29]: The Camouflaging Autistic Traits Questionnaire (CAT-Q) is a self-administered test composed of 25 items that concern social masking behaviors. It is used to identify individuals who may not immediately exhibit ASD traits due to their ability to mask them. This may be particularly relevant for women with autism. It is characterized by the following subscales: (1) Compensation: strategies used to actively compensate for difficulties in social situations; (2) Masking: strategies used to hide autistic symptomatology or portray a non-autistic personality; and (3) Assimilation: strategies used to try to integrate with others in social situations.
- CASD (Checklist for Autism Spectrum Disorder) [30]: The CASD consists of 20 questions assessing the children and adolescents’ behaviors in several areas, including communication, social interaction, imagination, and repetitive behaviors. The questions are divided into four categories: “Communication”, “Socialization”, “Repetitive Behaviors”, and “Imagination”. The answers to the questions are rated on a scale of 0 to 2 depending on the intensity of the behavior observed, and the sum of the scores determines whether the child is at high, moderate, or low risk of having an ASD.
- Toronto Alexithymia Scale (TAS–20) [31]: The TAS-20 is the most used tool to evaluate alexithymia. This 20-item questionnaire has 3 factorial scales: (1) DIF (Difficulty Identifying Feelings): difficulty identifying feelings and distinguishing between feelings and physical sensations; (2) DDF (Difficulty Describing Feelings): difficulty describing one’s feelings to others; and (3) EOT (Externally Oriented Thinking): cognitive style oriented towards external reality.
- Eating Disorder Inventory—3 (EDI-3) [32]: A self-assessment questionnaire, known as EDI-3, is routinely utilized for diagnosing clinically relevant symptoms of FED. Comprising 91 items across 12 scales, 3 scales cater specifically to FED (Drive for Thinness—DT; Bulimia—B; and Body Dissatisfaction—BD), while the remaining 9 assess general psychological symptoms (Low Self-Esteem—LSE; Personal Alienation—PA; Interpersonal Insecurity—II; Interpersonal Alienation—IA; Interoceptive Deficits—ID; Emotional Dysregulation—ED; Perfectionism—P; Asceticism—A; and Maturity Fears—MF) pertinent to FED.
- The Eating Disorder Examination Questionnaire (EDE-Q) [33]: The EDE-Q is a 28-item questionnaire that investigates attitudes and behaviors related to nutrition and eating disorders. The EDE-Q consists of a rating scale of choice with a score ranging from 0 to 6, with scores equal to or greater than 4 indicatives of the clinical range. The included subscales are Dietary Restraint, Eating Concern, Shape Concern, and Weight Concern.

The use of well-defined cut-offs in the proposed tests ensures that the results are comparable with other studies and can be easily integrated into the scientific literature. They also reduce the possibility of including participants with marginal or subclinical characteristics that might confound the results, ensuring a more homogeneous study cohort and thus enabling more precise conclusions to be formulated. Only cut-offs adopted by official tests and cited reference articles were adopted.

The following definitions were adopted for this study:

Diagnosis of ASD: For the purposes of this study, a “diagnosis of ASD” is defined as a diagnosis of Autism Spectrum Disorder made according to the diagnostic criteria of the DSM-5. Such diagnosis is made based on clinical judgment in light of cumulative evidence from all assessments performed as per clinical practice; a diagnosis operatively required a present ADOS-2 score for “autism” or “autism spectrum” and a CASD score indicative of an early developmental history of ASD traits.

ASD Traits: For this study’s purposes, “ASD traits” are defined as evidence of scores above threshold on one or more of the following tests, understood as total score or subscale (as per clinical practice). Therefore, diverse types of ASD traits could be identified, detected by distinct tests as follows:

Alexithymia according to the TAS scale:

○ Deficits in social skills, cognitive flexibility, attention to detail, and communication according to the AQ scale;
○ Personal experience of social masking of autistic symptoms according to the CAT-Q scale;
○ Current ASD symptoms observed clinically in real time according to the ADOS-2 test.

### 2.4. Statistical Analysis

Descriptive analyses were conducted for the whole sample. A significance level of 0.05 was established for all statistical tests, and two-tailed tests were employed. To evaluate the normality of data distribution and the homogeneity of variance, Shapiro–Wilk’s and Levene’s tests were utilized. The two groups were subsequently compared using the chi-squared test for categorical variables (with the Fisher exact test being employed when necessary due to small sample sizes) and the Student’s *t*-test for continuous variables (Mann–Whitney U Test was used for non-normally distributed data). Differences between included diagnostic groups Restrictive-AN (AN-R), Binge-Purging-AN (AN-BP), and AAN were assessed with analysis of variance (ANOVA). Correlations between continuous variables (EDI-3 Interoceptive Deficits on one side; ASD traits measured with AQ, TAS-20, CAT-Q, and ADOS-2 on the other side) were measured using Pearson’s r (Spearman’s rho was needed). Finally, a linear regression was conducted to identify potential predictors for EDI-3 Interoceptive Deficits, among ASD traits potentially correlated with this variable. The determination of the sample size was based on the number of individuals enrolled during the study period. Given the observational design of this study focusing on the naturalistic portrayal of real-world data, missing data were not imputed or replaced. All the statistical analyses were carried out using JASP (Jeffrey’s Statistical Program), specifically version 17.1 for Windows.

## 3. Results

### 3.1. Clinical and Sociodemographic Characteristics

Until now, of the 53 patients diagnosed with AN/AAN who were invited to participate in the study and met the inclusion criteria, 52 agreed to undergo the assessment with their parents. The recruited patients’ mean Body Mass Index (BMI) was 16.7 kg/m^2^. The most frequently represented diagnoses were AN, Restrictive subtype and AAN. From the anamnestic collection, it was found that four patients (7.5%) had a family history of an FED, six (11.3%) a Mood Disorder, four (7.5%) an anxiety disorder, one (1.9%) an intellectual disability, one (1.9%) a substance use disorder, and one (1.9%) a learning disorder. None of the patients had a family history of ASD.

Three quarters of the patients included (75.0%) were receiving a second-generation antipsychotic, while 53.9% were receiving a selective serotonin reuptake inhibitor. At least one month passed for each included patient since the last psychopharmacological therapy had been introduced. The descriptive data for the entire sample are presented in Table 1.

### 3.2. Autism Spectrum Disorder Traits

The obtained scores for ASD traits are reported in Table 2. Five patients exhibited an ADOS-2 score indicative of an ASD diagnosis (9.6%). Above-average scores compared to neurotypical controls were evident in specific domains of the AQ, particularly in AQ—Social Skills (3.65), AQ—Cognitive Flexibility (5.42), AQ—Communication (4.42), and AQ—Imagination (3.35). Only eight patients (15.4%) reported clinical scores on the administered CASD, as reported by their parents.

### 3.3. Assessment of Alexythimia and Camouflaging of ASD Traits

The scores concerning the assessment of alexithymia (TAS-20) and camouflaging of ASD traits (CAT-Q) are reported in Table 3. An above-average score was observed in the CAT-Q Assimilation area (32.86), with 22 patients (42.3%) showing a clinical score in this domain. In assessing alexithymia using the TAS (Toronto Alexithymia Scale), we observed clinically significant average scores in both the total score and the three subscales.

### 3.4. Differences Between AN Diagnostic Groups

No statistically significant differences were documented when comparing the three AN diagnostic groups (AN-R, AN-BP, and AAN) concerning TAS total score (*p* = 0.420), CAT-Q total score (*p* = 0.544), CASD score (*p* = 0.576), AQ total score (*p* = 0.427), or the final classification obtained regarding the ADOS-2 (*p* = 0.315).

### 3.5. Assessment of Eating Disorder Symptomatology

Significant average scores are evident regarding the variables EDI-3—Inadequacy (75.65), EDI-3—General Psychological Maladjustment (78.92), and EDI-3—Affective Problems (78.38). All the variables from the Body Attitudes Questionnaire and the Body Uneasiness Test exhibit average scores above the norm. Increasing scores in these variables correspond to a higher severity of psychopathological symptoms associated with the ED. Mean scores for ED symptomatology are reported in Supplementary Material Table S1.

### 3.6. Correlations Between Interoceptive Deficits, ASD Traits, Alexithymia, and Camouflage

Supplementary Material Table S2 reports the existing correlations between EDI-3 Interoceptive Deficits and ASD traits, alexithymia, and camouflage. EDI-3 Interoceptive Deficits were positively correlated to AQ—total score (p=0.004), AQ—attention switching (*p* = 0.009), CAT-Q—Compensation (*p* = 0.022), TAS—total score (*p* = 0.017), TAS—Difficulty Identifying Feelings (*p* < 0.001), and TAS—Difficulty Describing Feelings (*p* = 0.008). EDI-3 Interoceptive Deficits also negatively correlated with ADOS-2 social interaction (*p* = 0.047).

### 3.7. Predictive Model for EDI-3 Interoceptive Deficits

**Figure 1:**
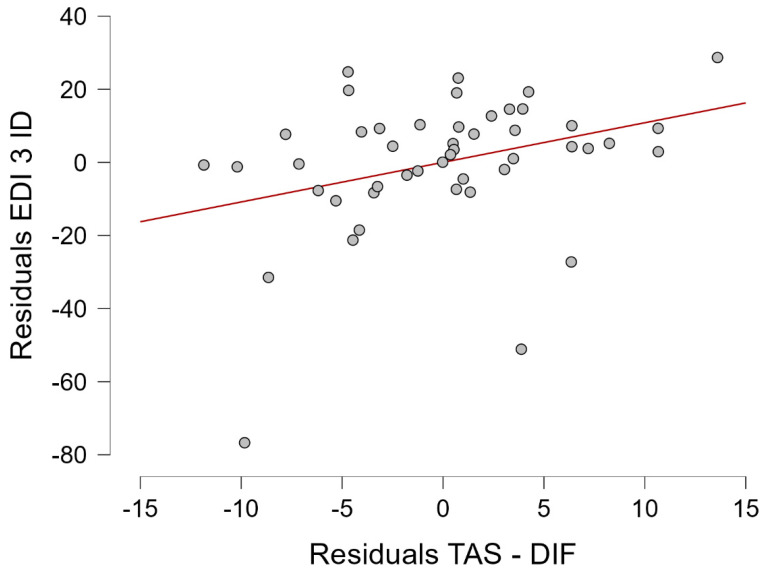
Residuals for TAS—Difficulty Identifying Feelings regarding a linear regression assessing potential predictors for EDI-3 Interoceptive Deficits.

A linear regression assessing the potential predictors for EDI-3 Interoceptive Deficits documented a predictive model (F(5,46) = 2.756; *p* = 0.031; R^2^ = 0.252). After the regression process, TAS—Difficulty Identifying Feelings was retained as the sole predictor for EDI-3 Interoceptive Deficits (*p* = 0.030). The residuals for this linear regression are reported in Figure 1.

## 4. Discussion

The assessment of the overlapping conditions between AN and ASD represents a crucial aspect of clinical practice. Remarkably, 9.6% of the patients included in the present preliminary retrospective research presented an ADOS-2 clinical impression consistent with a diagnosis of ASD. This finding aligns with recent research indicating overlapping ASD diagnoses in female AN patients, suggesting delayed ASD diagnoses based on nutritional and ED symptoms. Longitudinal studies support the notion that ASD traits, especially in social interaction, precede adolescent FEDs independently from BMI [26,34]. The systematic review conducted by Boltri et al. and based on the analysis of 13 studies suggests that comorbidity between AN and ASD may exacerbate the severity of the anorexic condition, with the ASD traits appearing to be stable over time and tending to persist after weight recovery [35]. Despite only eight patients in our sample showing risk scores for ASD in the parent-administered CASD interviews, three of them presented an ADOS-2 clinical impression consistent with an ASD diagnosis. Notably, two patients exhibiting ASD had clinical scores exceeding the neurotypical norms in CAT-Q, particularly in compensation, masking, and assimilation.

The study’s clinical sample displayed an overall CAT-Q score within the norm. Still, it showed a clinically elevated score in the Assimilation subscale, indicating how AN patients utilize these strategies in relational contexts. This aligns with the literature suggesting that these strategies support FED symptoms as a means of assimilation and peer connection [36]. The literature underlines the adaptive challenges of such social strategies in autism diagnosis, particularly in females [3]. Girls, adept at integrating verbal and non-verbal communication, exhibit greater imaginative capacities but struggle to sustain friendships, facilitating their unnoticed presence in clinical settings for extended periods [37].

Interoceptive awareness and interoceptive deficits represent a major theme in the recent research conducted on individuals with AN. Compared to controls, patients with AN show reduced interoceptive awareness [38]. Hypoconnectivity between subcortical–cortical midline structures, a neural correlate of reduced intero-exteroceptive integration, has also been documented [39]. In the present study, interoceptive deficits were found to correlate with measures of ASD traits, alexithymia, and camouflage of ASD symptoms. The research indicates that individuals with ASD often face challenges in perceiving and integrating physiological feedback from their bodies. However, studies have also proposed that there may not be a significant difference in interoceptive ability between individuals with and without ASD once alexithymia is taken into consideration [40]. Alexithymia, defined by difficulty in recognizing and describing emotions, involves a broader cross-cutting problem related to interoception as a whole, the latter being fundamental to emotional recognition and integrated body awareness. The interoceptive deficit in alexithymia could result either from reduced sensitivity to body signals, which limits the ability to perceive internal information, or from the dysfunction and integration of these signals. This dysfunction, which involves specific neural circuits, such as the insula and anterior cingulate cortex, prevents the use of body feedback signals to modulate emotions and behaviors, thus impairing body awareness [23].

The differential correlations between specific ASD traits and eating disorder symptoms in our study may reflect distinct underlying mechanisms. Social communication difficulties, as assessed by measures like the ADOS-2 and AQ, may primarily influence symptoms such as interpersonal insecurity or emotional dysregulation given their shared reliance on social interaction and emotional understanding. In contrast, traits like cognitive inflexibility and alexithymia likely have a stronger impact on interoceptive deficits and rigid dietary behaviors, which are more related to internal processes and difficulties in adapting to change.

Relevantly, when performing a linear regression analysis, alexithymia (particularly the “Difficulty Identifying Feelings” subscale) emerged in our study as the sole predictor for interoceptive deficits in young individuals with AN. The intersection of alexithymia and interoceptive deficits emerges as a pivotal nexus between ASD and AN [21,22]. In a recent study adopting a network analytic approach, TAS-20—Difficulty Identifying Feelings stands out as the strongest domain of alexithymia associated with interoception in a non-clinical sample [41]. As a recent study highlighted, interoception study in FEDs encounters obstacles; a fresh active inference paradigm links alexithymia to sensory-driven behavior, unveiling diminished precision and heightened prediction errors. We think that our data expand the presently available literature on this topic, documenting that alexithymia may represent the potential link to explain the occurrence of interoceptive deficits in individuals with AN and ASD traits.

This may have direct therapeutic implications. A recent study on mindfulness, for instance, suggests that heightened alexithymia could correlate with varying interoceptive accuracy, although meditation did not significantly enhance the interoceptive accuracy or sensitivity compared to a control group [42].

Despite the limitations, including a relatively small sample size and the cross-sectional design of the study, which constrains the ability to infer causal relationships between the analyzed variables as it offers a static depiction at a single point in time, this study contributes valuable insights regarding the interplay between ASD traits and AN. This research describes preliminary data that may be the basis for broader future prospective multicentric research that will enhance real-time data collection, offering temporal insights into the relationship consequently determining whether the ASD should be considered as a risk factor or a consequence of an FED.

Ethical adherence to the Helsinki Declaration standards underscores research integrity. Comprehensive neuropsychiatric assessments strengthen the study’s depth. The inclusion criteria, such as the elapsed time since psychopharmacological therapy, a specific age range, and a BMI > 1400 Kg/m^2^ ensure sample homogeneity. The multifaceted approach, examining ASD traits, interoceptive deficits, and ED symptomatology, provides a comprehensive understanding of coexisting AN and ASD traits. While the generalizability may be limited, the study’s strengths lie in its design, use of extensive assessments, contributions to the broader understanding of this complex intersection, and its ethical considerations.

Future research should aim to deepen the understanding of the complex relationships between ASD traits, interoceptive deficits, and AN. A primary focus could be on how the ability to perceive and process internal bodily signals contributes to disordered eating behaviors in individuals with ASD traits. Understanding how these interoceptive deficits manifest in individuals with ASD traits and how they influence eating patterns could help to clarify why these individuals are more prone to developing AN. In addition, neuroimaging studies exploring the connectivity between the brain regions involved in interoception and those responsible for regulating eating behaviors could shed light on the shared neurobiological mechanisms. By examining the neural networks underlying both body awareness and emotional regulation, the research could reveal how disruptions in these systems contribute to both ASD and AN. These findings may identify potential biomarkers for early detection and intervention in individuals at risk for both conditions.

Therapeutic interventions targeting interoception could be a crucial area of future research. Techniques such as mindfulness-based practices, interoception training, or sensory integration therapy may help individuals with ASD and AN to better regulate and interpret bodily signals. These interventions could reduce the anxiety associated with eating and body image, potentially improving the treatment outcomes for individuals with AN, particularly those on the ASD spectrum. Moreover, therapies aimed at improving emotional regulation and reducing anxiety could be tested for their effectiveness in addressing the unique challenges faced by individuals with both disorders.

Lastly, exploring the genetic and environmental factors contributing to the co-occurrence of ASD and AN could provide a deeper understanding of their shared vulnerabilities. Identifying specific genetic markers or environmental triggers, such as early sensory experiences, might reveal predispositions that make certain individuals more susceptible to both conditions. Understanding these factors could lead to more personalized treatment approaches, including preventive measures for individuals at risk.

## 5. Conclusions

In conclusion, our preliminary study reveals a noteworthy comorbidity of ASD in adolescent females with AN. Approximately 9.6% of the AN patients exhibited ASD traits, emphasizing the need for a nuanced understanding of the overlapping symptoms. The assessment of interoceptive deficits, ASD traits, and camouflaging strategies underscores intricate connections. TAS-20—Difficulty Identifying Feelings emerged as a critical predictor for interoceptive deficits in individuals with AN and ASD traits. Early identification and intervention strategies, incorporating comprehensive assessments, are crucial for addressing the complex interplay between AN and ASD. Recognizing camouflaging strategies sheds light on the adaptive challenges faced by individuals, particularly females, integrating into social contexts while exhibiting ASD traits. This study highlights the intersection of alexithymia and interoceptive deficits as a pivotal nexus between ASD and AN, offering insights for targeted therapeutic interventions. Continued research is warranted to enhance the clinical management and intervention strategies for this overlapping population.

## Figures and Tables

**Table 1:** Clinical characteristics of the enrolled sample. Continuous variables are presented as mean ± standard deviation; nominal variables are presented as number (%).

Variables	Data (n = 52)
Female gender	52 (100%)
BMI (kg/m^2^)	16.8 ± 1.6
%BMI (%)	82.9 ± 7.9
Average Age (years)	15.3 ± 1.4
Diagnosis	AN-R = 28 (53.9%)
	AAN = 20 (38.5%)
	AN-BP = 4 (7.7%)
Family History of a mental health condition	16 (30.7%)
Pharmacological Therapy	SSRI = 28 (53.9%)
	SGA = 39 (75.0%)
Comorbidities	Depressive traits = 5 (9.6%)
	Anxiety traits = 7 (13.5%)
	Transitory motor tics = 1 (1.9%)

Abbreviations: AAN: Atypical Anorexia Nervosa; AN-BP: Anorexia Nervosa, Binge-Purging subtype; AN: Anorexia Nervosa, Restrictive subtype; BMI: Body Mass Index; SGA: second-generation antipsychotics; SSRIs: selective serotonin reuptake inhibitors.

**Table 2:** Assessment of ASD traits.

Variables	Under the Clinical Threshold (n, %)	Above Clinical Threshold (n, %)	Mean (SD)
ADOS -2—Overall Impression	47 (90.4)	5 (9.6)	/
ADOS-2—Communication	42 (80.8)	10 (19.2)	0.71 (1.36)
ADOS-2—Social Interaction	43 (82.7)	9 (17.3)	1.12 (1.80)
ADOS-2—Imagination/Creativity	27 (51.9)	25 (48.1)	0.51 (0.54)
ADOS-2—Communication + Social Interaction	47 (90.4)	5 (9.6)	1.80 (3.12)
AQ—Total	48 (92.3)	4 (7.7)	19.46 (9.35)
AQ—Social Skills	21 (40.4)	31 (59.6)	3.65 (2.33)
AQ—Cognitive Flexibility	13 (25.0)	39 (75.0)	5.42 (1.9)
AQ—Attention to Details	37 (71.2)	15 (28.8)	4.48 (2.24)
AQ—Communication	16 (30.8)	36 (69.2)	4.42 (2.76)
AQ—Imagination	14 (26.9)	38 (73.1)	3.35 (1.96)
CASD	44 (84.6)	8 (15.4)	4.23 (3.15)

**Table 3:** Assessment of alexithymia and camouflaging of ASD traits.

Variables	Under the Clinical Threshold (n, %)	Above Clinical Threshold (n, %)	Mean (SD)
CAT-Q—Total	36 (69.2)	16 (30.8)	85.5 (24.3)
CAT-Q—Compensation	32 (61.5)	20 (38.5)	24.9 (10.2)
CAT-Q—Masking	34 (65.4)	18 (34.6)	30.42 (8.65)
CAT-Q—Assimilation	30 (57.7)	22 (42.3)	32.86 (13.5)
TAS—Total	9 (17.3)	43 (82.7)	63.11 (13.02)
TAS—Difficulty Identifying Feelings	7 (13.5)	45 (86.5)	26.70 (7.85)
TAS—Difficulty Describing Feelings	5 (9.6)	47 (90.4)	18.26 (4.4)
TAS—Externally Oriented Thinking	18 (34.6)	34 (65.4)	20.50 (6.07)

## Data Availability

Data will be available from the corresponding author upon reasonable request.

## Supplementary Materials

The following supporting information can be downloaded at https://www.mdpi.com/article/10.3390/pediatric16040092/s1, Table S1: Eating disorder symptomatology; Table S2: Correlations (table and related heatmap) between EDI-3 Interoceptive Deficits, ASD traits, alexithymia, and camouflaging.
