# Supplementary material for: Autism Spectrum Disorder Traits Predict Interoceptive Deficits and Eating Disorder Symptomatology in Children and Adolescents with Anorexia Nervosa—A Cross-Sectional Analysis: Italian Preliminary Data
Source: Pediatr Rep. 2024 Dec 5;16(4):1077–88. doi: 10.3390/pediatric16040092 (PMC11676964; doi:10.3390/pediatric16040092)
Supplement: Supplementary file 1 [file pediatrrep-16-00092-s001.zip › pediatrrep-3317017-supplementary.pdf]

Supplementary Material Table S1. Eating Disorder Symptomatology

Variables	Mean (SD)
EDE-Q Restraint	3,53 (3,034)
EDE-Q ShapeConcern	4,60 (2,13)
EDE-Q Weight Concern	3,17 (2,47)
EDE-Q EatingConcern	3,18 (1,88)
EDI 3-Eating Disorder Risk Composite (EDRC)	66,25 (27,02)
EDI 3-Ineffectiveness (IC)	**75,65 (20,14)**
EDI 3-Interpersonal Problems (IPC)	68,21 (20,22)
EDI 3-Affective Problems (APC)	**78,38 (17,56)**
EDI 3-Overcontrol (OC)	67,73 (25,45)
EDI 3-General Psychological Maladjustment (GPMC)	**78,92 (17,40)**

Note: clinically relevat scores are reported in bold.

Supplementary Material Table S2. Correlations (Table and related Heatmap) between EDI-3 Interoceptive deficits, ASD traits, Alexythimia, and Camouflaging

	**Spearman's rho**	**p**
AQ - Total	0.411	**0.004 ***
AQ - Social Skills	0.183	0.214
AQ - Attention Switching	0.374	**0.009 ***
AQ - Attention to Detail	0.220	0.133
AQ - Communication	0.171	0.244
AQ - Imagination	0.135	0.359
ADOS-2 Social Interaction	-0.280	**0.047 ***
ADOS-2 Communication	-0.178	0.211
ADOS-2 Imagination / Creativity	-0.175	0.219
ADOS-2 StereotypedBehaviors	/	/
CASD	-0.033	0.814
CAT-Q - Total	0.126	0.383
CAT-Q - Compensation	0.323	**0.022 ***
CAT-Q - Masking	0.110	0.448
CAT-Q - Assimilation	0.154	0.284
TAS - Total	0.329	**0.017 ***
TAS - DifficultyIdentifying Feelings	0.450	**< .001 ***
TAS - DifficultyDescribing Feelings	0.366	**0.008 ***
TAS - ExternallyOriented Thinking	0.033	0.816

Note: significant correlations are reported in bold and with an asterisk.

ASD variables	EDI-3 Interoceptive Deficits		ASD variables
AQ - Total	0.411	0.126	CAT-Q - Total
AQ - Social Skills	0.183	0.323	CAT-Q - Compensation
AQ - Attention Switching	0.374	0.110	CAT-Q - Masking
AQ - Attention to Detail	0.220	0.154	CAT-Q - Assimilation
AQ - Communication	0.171	0.329	TAS - Total
AQ - Imagination	0.135	0.450	TAS – Difficulty Identifying Feelings
ADOS-2 Social Interaction	-0.280	0.366	TAS – Difficulty Describing Feelings
ADOS-2 Communication	-0.178	0.033	TAS – Externally Oriented Thinking
ADOS-2 Imagination / Creativity	-0.175	-0.033	CASD
ADOS-2 Stereotyped Behaviors	/		
